# Isolation and Characterization of Adhesive Secretion from Cuvierian Tubules of Sea Cucumber *Holothuria forskåli* (Echinodermata: Holothuroidea)

**DOI:** 10.1155/2011/486845

**Published:** 2011-06-15

**Authors:** Malgorzata Baranowska, Ute Schloßmacher, J. Douglas McKenzie, Werner E. G. Müller, Heinz C. Schröder

**Affiliations:** ^1^Institute for Physiological Chemistry, University Medical Center of the Johannes Gutenberg University, Duesbergweg 6, 55128 Mainz, Germany; ^2^Xanthella Ltd., Marine Resource Centre, Barcaldine, Argyll, Scotland PA37 1SG, UK

## Abstract

The sea cucumber *Holothuria forskåli* possesses a specialized system called Cuvierian tubules. During mechanical stimulation white filaments (tubules) are expelled and become sticky upon contact with any object. We isolated a protein with adhesive properties from protein extracts of Cuvierian tubules from *H. forskåli*. This protein was identified by antibodies against recombinant precollagen D which is located in the byssal threads of the mussel *Mytilus galloprovincialis*. To find out the optimal procedure for extraction and purification, the identified protein was isolated by several methods, including electroelution, binding to glass beads, immunoprecipitation, and gel filtration. Antibodies raised against the isolated protein were used for localization of the adhesive protein in Cuvierian tubules. Immunostaining and immunogold electron microscopical studies revealed the strongest immunoreactivity in the mesothelium; this tissue layer is involved in adhesion. Adhesion of Cuvierian tubule extracts was measured on the surface of various materials. The extracted protein showed the strongest adhesion to Teflon surface. Increased adhesion was observed in the presence of potassium and EDTA, while cadmium caused a decrease in adhesion. Addition of antibodies and trypsin abolished the adhesive properties of the extract.

## 1. Introduction

Adhesion plays an important role in many invertebrates for a variety of different functions. Some species of holothuroid echinoderms (sea cucumbers) possess a special defence system involving adhesion, based on secretion of highly adhesive filaments that can entangle predators. This system called Cuvierian tubules is mainly activated when animals are mechanically stimulated but may also be stimulated by heat. As a result sea cucumbers release white filaments, tubules, which become sticky upon contact with any object [1–5]. 

In the search for potential technological applications understanding the chemistry of the adhesive secretion from Cuvierian tubules is important. Applications may include the design of water-resistant adhesives, sealants, and biomedical coatings and the development of new antifouling strategies [6, 7].

The amazing adhesivity of the Cuvierian tubule filaments was investigated by Müller et al. [8], Zahn et al. [9], and Flammang et al. [2], whereby tubules were treated with various substances and adhesion to different surfaces was measured. However, protein extracts from Cuvierian tubules have not yet been investigated for their adhesion properties. Many researchers have studied the histochemistry of Cuvierian tubules, as early as in 1868 (Semper [10], followed by Jourdan [11], Hérouard [12], and later Müller et al. [13]).

The fine structure of Cuvierian tubules was investigated by VandenSpiegel and Jangoux [14]. Quiescent Cuvierian tubules consist of an outer mesothelium, an inner epithelium, and between them, a thick connective tissue layer that includes muscle fibres. Biochemical investigations by DeMoor et al. [5] revealed that Cuvierian tubules are made up of 60% protein and 40% carbohydrates. They are highly insoluble. 

Antibodies raised against the material that remained on the substratum after detachment of the tubule have been used to detect and localize tubule protein(s) [5].

Also the adhesion of mussels has been thoroughly investigated. After isolation of the first adhesive protein, Mepf-1, which contains DOPA (3,4-dihydroxy-l-phenylalanine), ten further proteins have been isolated [7]. One of them is precollagen D which possesses a central collagen domain flanked by two fibroin-like domains with sequences similar to spider silk fibroin. This protein is found in spiders' drag line [15] and also in the silkworm *Bomyx mori* [16]. A similar protein has been found in sea urchin [17]. Using antibodies raised against recombinant precollagen D from the mussel *Mytilus galloprovincialis*, we could identify a protein with adhesive properties in sea cucumber Cuvierian tubule extracts.

## 2. Materials and Methods

### 2.1. Sea Cucumber

Sea cucumber *Holothuria forskåli *was collected at the coast of the Adriatic Sea near Rovinj (Istria, Croatia) with a dragnet at a depth 20 m in August 2007. Specimens were anaesthetized for 1 h in a saturated solution of urethane (Sigma-Aldrich, Taufkirchen, Germany) and then transported in methanol at 4°C to the laboratory before Cuvierian tubules were removed.

### 2.2. Extraction of Cuvierian Tubules

Cuvierian tubules were dried by lyophilization and ground in a mortar containing liquid nitrogen. Dried material (2 g) was stirred with 50 mL of buffer (4 M urea, 0.5 M Tris-HCl, pH 7.5) overnight at 4°C. The homogenate was then centrifuged at 14,000 ×g for 15 min. The supernatant was collected, filtered through 2-*μ*m filter (Centrifugal Filter Devices Microcon, Millipore, Schwalbach, Germany) and dialyzed overnight against 5 l of distilled water which was changed every 3 h. To concentrate the sample, the dialysate was centrifuged (4.000 ×g) in a concentration tube (50 mL) using a centrifugal concentrator (Amicon, Millipore, Schwalbach, Germany) until 1 mL of extract was obtained.

### 2.3. SDS-PAGE and Western Blot Analysis

Extract from Cuverian tubules was concentrated with Ready Prep 2-D Clean-up Kit (Bio-Rad, Munich, Germany). The concentrated protein was dissolved in loading buffer (Roti-Load, Roth, Karlsruhe, Germany), boiled for 5 min and subjected to electrophoresis in a 12% polyacrylamide gel containing 0.1% sodium dodecyl sulfate (SDS-PAGE). After protein separation the gel was washed with distilled water for 15 min and then stained with Gel Code Blue Reagent (Pierce, Bonn, Germany).

For Western blot analysis, proteins were transferred from the gels to PVDF membranes (Millipore, Schwalbach, Germany) using a Trans-Blot SD system (Bio-Rad, Munich, Germany). The membranes were blocked with Blocking reagent (Roche, Mannheim, Germany), then rinsed in TBS-T (20 mM Tris-HCl pH 7.6, 137 mM NaCl, 0.1% Tween-20) and incubated for 1 h with polyclonal antibodies (rabbit) that had been raised against precollagen D from mussel *M. galloprovincialis *(PoAb-pCD). The dilution of the antiserum was 1 : 1000. The membrane was washed three times in TBS-T and then incubated for 1 h with anti-rabbit IgG (whole molecule) alkaline phosphatase (Sigma-Aldrich, Taufkirchen, Germany). After washing, proteins were visualized using 5-bromo-4-chloro-3-indolyl phosphate-*p*-toluidine salt (BCIP) and *p*-nitrotetrazolium blue chloride (NBT) (Roth, Karlsruhe, Germany).

### 2.4. Isolation of Adhesive Protein by Electroelution

The protein band from the electrophoresis gel which reacted with antibodies against precollagen D was cut from the gel and electroeluted using a Model 422 Electro-Eluter (Bio-Rad, Munich, Germany). The purity of the obtained protein was checked by SDS-PAGE and Western blot analysis and concentration was estimated by 2-D Quant kit (Amersham Biosciences, Freiburg, Germany).

### 2.5. Antibody Production

Polyclonal antibodies (PoAb) were raised against the protein obtained by electroelution by immunization of female rabbits (White New Zealand) as described [18]. After three boosts the serum was collected and screened in a conventional ELISA assay as well as by Western blotting.

### 2.6. Collection of Adhesive Proteins

Glass beads (1 g) with a size of 2 mm (Elring-Klinger Kunststofftechnik GmbH Grossostheim-Ringheim, Germany) were washed with 4 M urea buffer (containing 0.5 M Tris-HCl, pH 7.5) and added to each of four tubes containing the same amount of protein (500 *μ*g) but various concentrations of urea (1 M, 2 M, and 3 M). The tubes with the glass beads were incubated under shaking for 2 h and vortexed every 10 min. The supernatants were then discarded and the beads were washed with 0.05 M Tris-HCl pH 7.5 (1 mL) by shaking and vortexing. The washing step was repeated three times. The buffer was then removed and 50 *μ*L of sample buffer (4-times concentrated; 100 mM Tris-HCl, pH 6.8, 2% SDS, 5% ß-mercaptoethanol, 15% glycerol, 0.006% bromophenol blue) was added to each tube. The glass beads were then boiled and shaken for 15 min at 95°C in a thermomixer. Thereafter the supernatants were collected and 40 *μ*L each were taken for loading onto two 12% SDS PAGE gels. One gel was stained with Gel Code Blue Reagent and destained with water, then scanned with an Odyssey Scanner using the Odyssey v.1.2 software to quantify the protein bands. The proteins of the second gel were transferred to PVDF membrane and incubated with antibody against adhesive protein and developed using anti-rabbit IgG (whole molecule) alkaline phosphatase and visualized with NBT and BCIP.

### 2.7. Immunoprecipitation

For immunoprecipitation of the adhesive protein, the Seize × Immunoprecipitation Kit (Pierce, Bonn, Germany) was used; 50 *μ*L of polyclonal antibody against adhesive protein from Cuvierian tubules (PoAb-Ctub) and 250 *μ*L of Cuvierian tubule extract were applied. Results were checked by SDS PAGE and Western blotting.

### 2.8. Gel Filtration

Sephadex G50 (Pharmacia Fine Chemicals, Uppsala, Sweden) was used for preparation of a gel filtration column (1 cm × 15 cm). Calibration of the column was performed using a mixture of bovine serum albumin (BSA), silk fibroin, and carbonic anhydrase. The retention time of each sample was recorded. The presence of the protein in each fraction was checked by the Bradford assay [19].

Before loading the extract, the column was washed with 4 M urea buffer. One milliliter of the 4 M urea extract of the Cuvierian tubules was loaded onto the column. Protein was eluted using 4 M urea. Forty 500-*μ*L fractions were collected. The protein concentration in each fraction was assayed using the Bradford assay. Protein-containing fractions were loaded onto a 12% SDS PAGE gel. The gel was stained with Gel Code Blue Reagent, destained with water, and scanned with an Odyssey scanner.

### 2.9. Histology

#### 2.9.1. Preparation of Tissue Sections

Cuvierian tubule tissue was fixed in 2% paraformaldehyde in PBS pH 7.4 overnight. After washing in PBS buffer (pH 7.4) containing 6.8% sucrose at 4°C overnight, dehydration was performed in 100% acetone. During the first 5 minutes acetone was renewed several times. The last portion of acetone was left overnight. Infiltration of the samples was done using Technovit 8100 (Heraeus Kulzer, Hanau, Germany) according to the instructions of the manufacturer. After hardening, 3–10 *μ*m thick sections were prepared using a rotary microtome. Slices were mounted on silane-coated slides (Sigma, Taufkirchen, Germany) for histology and immunohistochemistry.

#### 2.9.2. Hematoxylin and Eosin Staining

After washing in PBS and in distilled water, tissue sections on slides were stained in hematoxylin solution for 5 min. After staining slides were washed in tap water for 25 min and in distilled water for 5 min. Sections were stained in eosin for 1 min. After washing in distilled water, sections were dehydrated with increasing concentrations of isopropanol (75–100%) for 1 min each. Before mounting, slides were washed in detergent Roti Clear (Roth, Karlsruhe, Germany). Slides were mounted in DPX (Sigma, Taufkirchen, Germany), covered with cover slips, and sealed with nail polish.

#### 2.9.3. Cason's Trichrome

Sections on slides were washed in PBS for 5 min, then placed in staining solution (1% orange G, 1.5% acid fuchsine, 0.5% aniline blue, and 1% phosphotungstic acid) for 5 min. After staining sections were washed in water for 5 min, dehydrated in solutions containing increasing concentration of ethanol, cleared in detergent (Roti Clear), mounted with DPX, and sealed with nail polish.

#### 2.9.4. Methylene Blue and Azure B

A mixture of 1% azure and 1% methylene blue was used. Before staining sections were warmed up to 70°C. A drop of the stain was put on the sections. After drying (60°C), unbound stain was washed out with distilled water. Slides were then dried, mounted with DPX, and sealed with nail polish.

### 2.10. Immunohistochemistry

Sections were kept overnight in 4% BSA in PBS. After washing with PBS, samples were incubated with polyclonal antibody against adhesive protein from Cuvierian tubules (PoAb-Ctub; dilution 1 : 100). Sections with antibody were kept in a humid chamber at room temperature for 2 h. After washing in PBS (2 × 10 min), sections were incubated with Cy3-conjugated goat anti-rabbit IgG (Dianova, Hamburg, Germany) (diluted 1 : 100) in the dark at 37°C for 120 min. After additional washing (2 × 10 min) in PBS, staining with DAPI (4′-6-diamidino-2-phenylindole; Sigma-Aldrich, Taufkirchen, Germany) for 30 min was performed to highlight the nuclei. After washing and mounting with the Gel/Mount (Fluorescent mounting medium, Dako, Hamburg, Germany), sections were inspected for immunofluorescence using an Olympus AHBT3 light microscope. Preimmune serum was used as a negative control.

### 2.11. Transmission Electron Microscopy

Electron immunogold labeling was performed with Cuvierian tubule samples treated with 0.1% glutaraldehyde/3% paraformaldehyde in phosphate buffer, pH 7.4 for 3 h at room temperature. The material was dehydrated in ethanol and embedded in LR-White resin. Sections (60-nm thick) were cut and blocked with bovine serum albumin in PBS and then incubated with the primary antibody against adhesive protein from Cuvierian tubules (PoAb-Ctub; 1 : 1,000 for 12 h at 4°C). In controls, preimmune serum was used. After three washes with PBS, 1% BSA, sections were incubated with a 1 : 100 dilution of the secondary antibody (1.4-nm nanogold anti-rabbit IgG; diluted 1 : 200) for 2 h. Sections were rinsed in PBS, treated with glutaraldehyde in PBS, washed, and dried. Subsequently, enhancement of immunocomplex detection was performed with silver as described by Danscher [20]. The samples were transferred onto coated copper grids and analyzed using a Tecnai 12 microscope (FEI Electron Optics, Eindhoven, Netherlands).

### 2.12. Measurement of Adhesion

**Figure 1:**
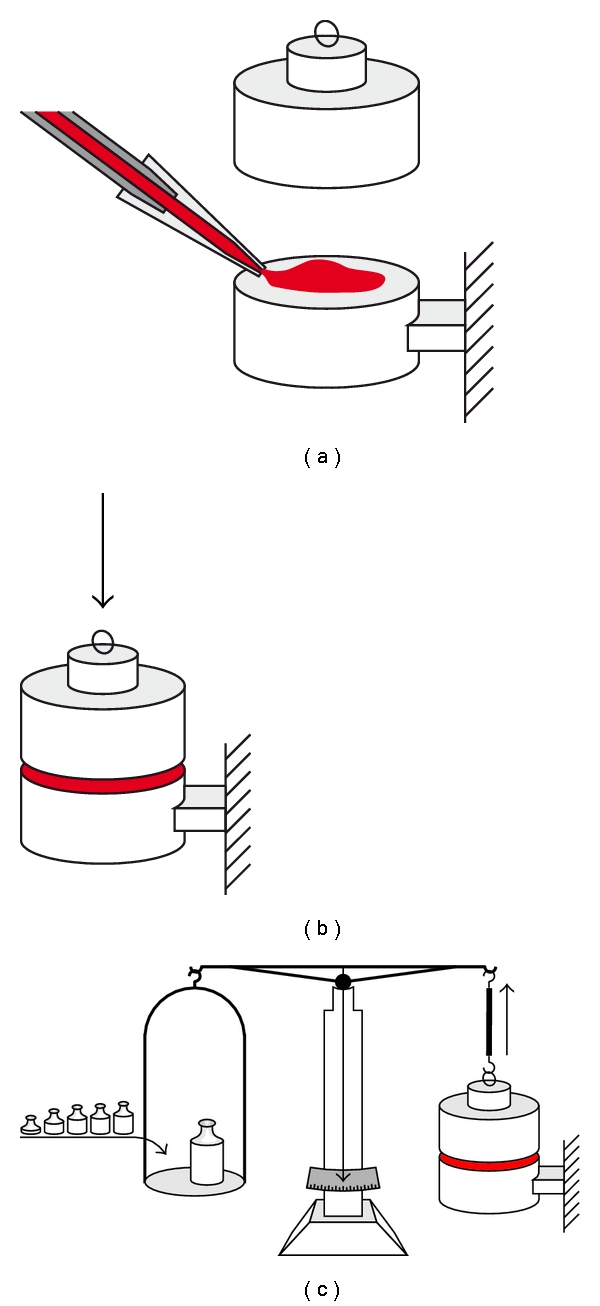
Scheme of equipment used for measurement of adhesion. (a) Application of the sample between two blocks; the upper block is attached to one of the beams of a laboratory balance, while the lower block is fixed. (b) Attachment of both blocks via the adhesive protein containing sample. (c) Addition of standard weights until both blocks become separated to determine the adhesive forces of the sample.

To measure adhesion, the instrument shown in Figure 1 was used. This instrument was based on a laboratory balance, modified by adding two blocks to it (made from either Teflon, iron, gelatine, glass, or silicone). The upper block was attached to one beam of the balance, while the lower block was attached to a movable platform. Adhesion was measured as follows: a drop of measuring liquid (10 *μ*L) was put on the block and stuck to the second block hanging from the beam. In this position, the drop of measuring liquid was incubated for 15 min at room temperature. To determine the adhesion of the liquid, standard masses were added until the two blocks separated when the adhesion of the liquid failed. The amount of weights put on, to release the block and liquid was equivalent to the adhesion forces between them.

### 2.13. Statistics

Statistical evaluation of the data was performed as described [21]. 

## 3. Results

### 3.1. Extraction of Cuvierian Tubules

**Figure 2:**
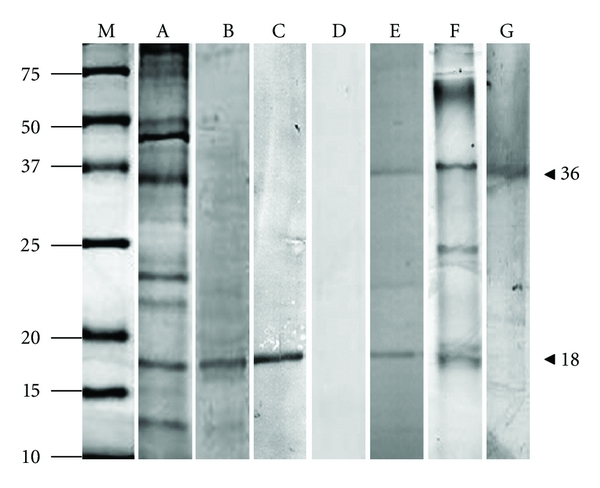
Analysis and identification of adhesive protein in Cuvierian tubule extract from sea cucumber* H. forskåli. *Proteins were analyzed by 12% SDS PAGE and Western blotting. Gels were stained with Gel Code Blue Reagent. (A) Cuvierian tubule extract (4 M urea, 0.5 M Tris-HCl pH 7.5) purified with Ready Prep 2-D Clean up kit. (B) Western blot of Cuvierian tubule extract, incubated with antibody against precollagen D (1 : 1000 dilution) and developed with anti-rabbit IgG alkaline phosphatase. (C) Western blot detection of adhesive protein from *H. forskåli*, isolated by electroelution, using antibody against precollagen D of mussel *M. galloprovincialis* (1 : 1000 dilution) (positive control). (D) Western blot of Cuvierian tubule extract incubated with preimmune serum. (E) Western blot of Cuvierian tubule extract incubated with antibodies against isolated (electroeluted) adhesive protein from *H. forskåli* (dilution 1 : 1000); the serum was purified by treatment with Cuvierian tubule extract (dilution 1 : 10) at a ratio of 1 : 5. (F) Cuvierian tubule extract, separated on seminative gel. (G) Western blot of Cuvierian tubule extract, separated on seminative gel (dilution of antibody, 1 : 1000); the serum was purified by treatment with Cuvierian tubule extract (dilution 1 : 10) at a ratio of 1 : 10. M: Molecular mass markers. *Arrowhead*s: adhesive protein (18 kDa) and dimer (36 kDa).

Several buffers were used for the extraction of protein from Cuvierian tubules. Protein solubilization was improved in basic rather than acidic buffers. Urea, SDS, and reducing buffers increased the extraction of tubule adhesive proteins. The amounts of protein extracted using various buffers were compared by SDS PAGE analysis. The best results were obtained using 4 M urea, 0.5 M Tris-HCl pH 7.5 (Figure 2, lane A), and sample loading buffer (100 mM Tris-HCl, pH 6.8, 2% SDS, 5% ß-mercaptoethanol, 15% glycerol, 0.006% bromophenol blue; results not shown). To improve visualization of the protein the extract shown in lane A was purified by the Ready Prep 2-D Clean-up kit. Other buffers like 150 mM NaCl, 1.5% NP_40_, 0.1% SDS, 0.1% DOC, 50 mM Tris-HCl pH 8 buffer or 0.5 M NaCl, 5 mM Tris-HCl pH 7.5, 7 mM Na_2_SO_4, _0.4 mM NaHCO_3,_ 20 mM EDTA buffer solubilized less protein (not shown). The 4 M urea buffer was used for further analysis because the composition of the sample loading buffer (see above) might interfere with further tests. 

SDS PAGE analysis of the purified Cuvierian tubule extract showed the presence of a wide size range of proteins (Figure 2, lane A). At this stage it was not possible to recognise which protein could be involved in adhesion. To get information about the conformation and possible dimerization/oligomerization of the proteins, the extract was run on a seminative gel (Figure 2, lane F). This method yielded a lower number of protein bands, indicating that some proteins might have been present as dimers/oligomers.

### 3.2. Identification of Adhesive Protein in Cuvierian Tubules

An antibody (PoAb-pCD; polyclonal antibody number N374) against a mussel byssus protein (recombinant precollagen D from *M. galloprovincialis*) was used to identify adhesive protein(s) of Cuvierian tubules. Precollagen D is a special collagen, found in the byssal thread, which is involved in tension-bearing. After reaction with the antibody the membrane showed a strong band (Figure 2, lane B) with one of the Cuvierian tubule proteins of a size 18 kDa. In further experiments this protein was recognized as the protein involved in adhesion. 

### 3.3. Isolation of Identified Protein

#### 3.3.1. Electroelution

The next step after the identification of the adhesive protein was its isolation. The band with a molecular mass of 18 kDa was cut out from the gel and electroeluted. The purity of the eluted protein was determined by SDS PAGE and Western blot analysis (Figure 2, lane C). The isolated protein was injected into a rabbit and after 4 weeks, the specific antibody designated PoAb-Ctub was obtained.

#### 3.3.2. Neutralization of Antibody

Western blot analysis of the crude extract of Cuvierian tubules using the PoAb-Ctub antibody revealed a strong reaction with several proteins of the extract even in the presence of optimized concentrations of antibody and extract (not shown).

The antibody was neutralized by incubation with Cuvierian tubule extract for 1-2 hours. After binding, the resulting mixture was applied onto the membrane of the Western blot. If the mixture contained an excess of specific antibodies, they would bind to the antigen on the membrane. 

The maximum increase in specificity of the antibody was observed after dilution to 1 : 1000 and addition of extract at a ratio of 1 : 5 (Figure 2, lane E). The Western blot showed two proteins which reacted with the antibody, one with a molecular mass of 18 kDa and the other of 36 kDa. Cuvierian tubule extract incubated with preimmune serum against the adhesive protein gave no reaction (Figure 2, lane D). We hypothesized that the upper protein band in lane E may represent a dimer of the lower protein band. In order to verify this, a native gel was run (Figure 2, lane F). After transfer of the protein, the membrane was incubated with the antibody against the adhesive protein. The results revealed the presence of only one band most likely caused by dimer formation of the adhesive protein (Figure 2, lane G).

#### 3.3.3. Immunoprecipitation

**Figure 3:**
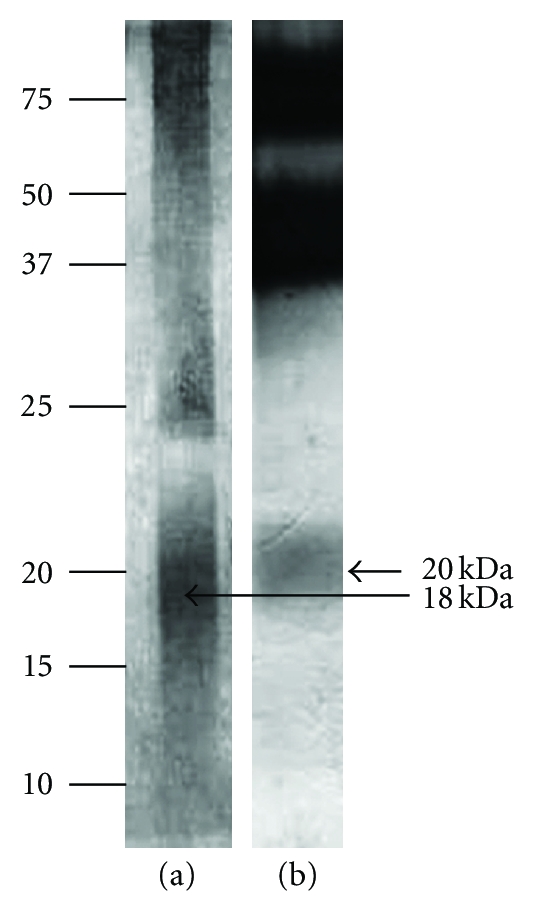
Western blot analysis of adhesive protein from Cuvierian tubule extract, purified by immunoprecipitation. The blot was developed using antibodies against adhesive protein from *H. forskåli* (PoAb-Ctub) and anti-rabbit IgG alkaline phosphatase secondary antibody. (a) Adhesive protein incubated with antibody against adhesive protein from *H. forskåli*. (b) Protein from mussels (precollagen D), incubated with antibody against adhesive protein from Cuvierian tubules. Numbers to the left indicate molecular masses of marker proteins in kDa.

Because the amount of adhesive protein obtained by electroelution was low and the buffer used for elution of the protein could complicate further analysis, other methods for isolation of the protein were employed. Immunoprecipitation is based on the interaction between a protein and its specific antibody, separation of the formed immune complex with Protein A, and subsequent Western blot analysis (Figure 3, lane (a)).

In a parallel experiment, extract from mussels *M. galloprovincialis* was applied on the immunocolumn with antibody against the adhesive protein from Cuvierian tubules. The result revealed a weak band at 20 kDa (Figure 3, lane (b)).

#### 3.3.4. Gel Filtration

Gel filtration chromatography (Sephadex G-50) was used for separation of the proteins based on their size. The fractions collected were assayed using the Bradford assay to detect the presence of protein. Protein-containing fractions were analyzed by SDS PAGE and measured for adhesion.

**Figure 4:**
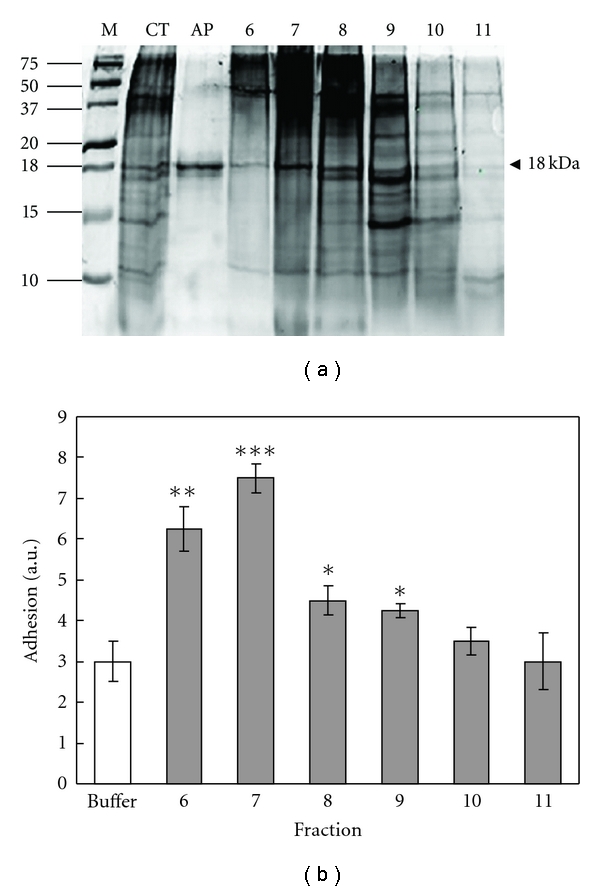
Analysis of fractions of gel filtration chromatography of Cuvierian tubule extract. (a) 15% SDS PAGE. *CT*: extract of Cuvierian tubules in 4 M urea, 0.5 M Tris-HCl buffer pH 7.5. *AP*: adhesive protein obtained by electroelution from Cuvierian tubule extract (positive control). *6–11*: fractions with highest concentration of protein according to Bradford assay.* M*, Molecular mass markers. *Arrowhead*: adhesive protein (18 kDa). (b) Adhesive activity, given in arbitrary units ± SD (*n* = 3), of fractions 6–11 compared to control (0.5 M urea buffer). Numbers to the left indicate molecular masses of marker proteins in kDa. Level of significance: *P < 0.05; **P < 0.01; ***P < 0.001.

The presence of adhesive protein was observed in fractions 6 to 8 (Figure 4). The strongest adhesion was found in fraction 7 (Figure 4(b)) where the concentration of adhesive protein was the highest (Figure 4(a)). Fractions 7 and 8 contained a higher degree of contamination by high-molecular-mass proteins. Relative molecular masses were determined using the equation: log⁡⁡*M* = *M*_0_ − (6.062 − 5.00 · *d*)  (*V*_*e*_/*V*_0_) (*M*, molecular mass; *d*, density of the swollen gel; *V*_*e*_, elution volume; *V*_0_, void volume) [22]. For the column used in the experiment shown in Figure 4, the equation is as follows: log⁡⁡*M* = 5.189 − 0.712  (*V*_*e*_/*V*_0_)  (see also [23]).

#### 3.3.5. Collection of Adhesive Proteins

**Figure 5:**
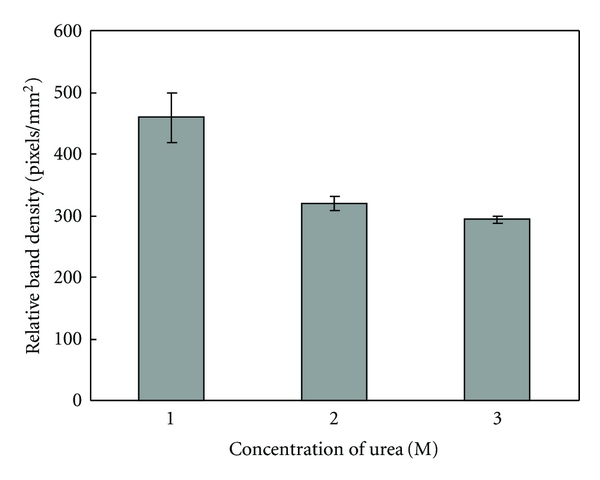
Binding of adhesive protein from Cuvierian tubule extract to glass beads in the presence of various concentrations of urea. Glass beads (size, 2 mm) were incubated with adhesive protein extract in the presence of various concentrations of urea (1 M, 2 M, and 3 M; containing 0.5 M Tris-HCl, pH 7.5), as described in Materials and Methods. After washing, the glass beads were boiled in SDS sample buffer and the released protein was analyzed by 12% SDS PAGE. The gel was stained with Gel Code Blue Reagent. Relative band intensities corresponding to the adhesive protein were determined by scanning of the gel using an Odyssey Scanner and applying the Odyssey v.1.2 software to quantify the protein bands.

Adhesive proteins may adhere to various surfaces. In this experiment adhesion to glass surface was used to isolate the *H. forskåli *protein from crude extract. Glass beads were incubated with protein extract containing the adhesive protein. After removing the extract and incubation of the glass beads with nondenaturing buffer, adhesive protein remained bound on the beads. To remove the protein from the glass surface, the beads were boiled with electrophoresis sample buffer and loaded onto 12% SDS PAGE. Relative band intensities corresponding to the adhesive protein were estimated using the Odyssey software. Proteins were transferred to PVDV membrane and analyzed by Western blot using the antibody against adhesive protein from Cuvierian tubules. The Western blots confirmed that the 18-kDa protein was the dominant protein harvested by using the glass beads procedure (not shown). Results after testing adhesion in the presence of various concentrations of urea revealed that a decrease in urea concentration of the extract resulted in an increased adhesion of the protein (Figure 5).

### 3.4. Histology

Cuvierian tubules were stained with Cason's trichrome, hematoxylin, and eosin, and methylene blue and azure to study their structure. Staining with these dyes showed that the quiescent tubules of *H. forskåli* consist of an outer mesothelium and an inner epithelium encompassing a thick connective tissue layer. The mesothelium is made of two cell layers, an upper layer of adluminal cells and a lower layer of granular cells.

#### 3.4.1. Cason's Trichrome

**Figure 6:**
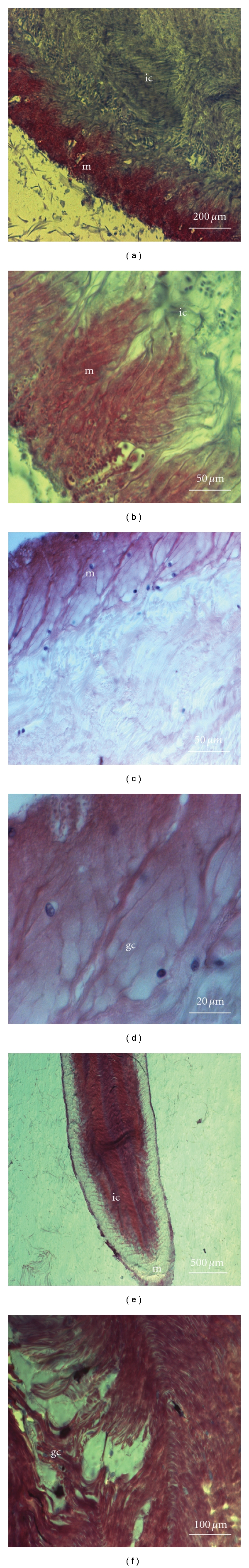
Sections of Cuvierian tubules stained with Cason's trichrome (a, b), hematoxylin and eosin (c, d), and methylene blue and azure B (e, f). *m*: mesothelium; *ic*: inner connective tissue; *gc*: granular cells.

This stain visualizes various organelles like nuclei (stained red) and collagen (blue). In sections of Cuvierian tubules, mesothelium was stained red, and the inner connective tissue was stained blue (Figures 6(a) and 6(b)).

#### 3.4.2. Hematoxylin and Eosin

Hematoxylin stains nuclei blue-purple. Eosin can be used to stain cytoplasm, collagen, and muscle fibers. In sections stained with hematoxylin and eosin, it was possible to observe the inner connective tissue layer stained red (eosin), while in the mesothelium the granular cells were stained purple (Figures 6(c) and 6(d)).

#### 3.4.3. Methylene Blue and Azure B

Methylene blue is used to visualize intracellular metachromatic granules. Azure, a methylated thiazine dye, is a metachromatic basic dye ranging from green (chromosomes) and blue (nuclei and cytoplasmic ribosomes) to red colour (deposits containing mucopolysaccharides).

Sections stained with methylene blue and azure B showed the presence of blue mesothelium and red inner connective tissue, while granular cells were stained dark (Figures 6(e) and 6(f)).

### 3.5. Immunostaining

**Figure 7:**
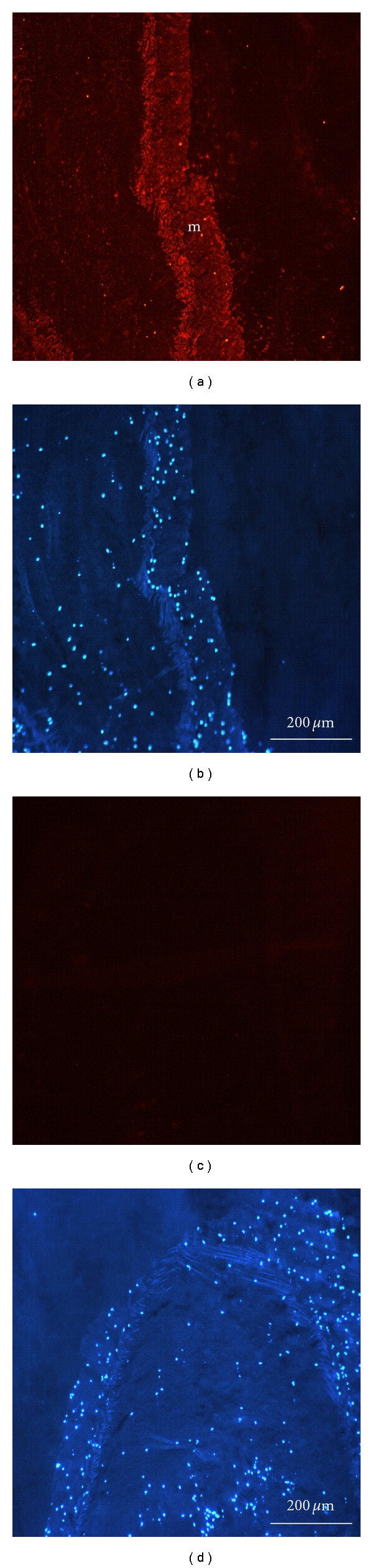
Immunohistological identification of adhesive protein in sections of Cuvierian tubules. (a, b) Sections of Cuvierian tubules stained with antibody against adhesive protein (PoAb-Ctub; 1 : 100 dilution (a) and counterstained with DAPI (b)). (c, d) Sections of Cuvierian tubules stained with preimmune serum (1 : 100; (c)) and counterstained with DAPI (d). Cy3-conjugated goat anti-rabbit IgG was used as secondary antibody. *m*: mesothelium.

The localization of adhesive protein in Cuvierian tubules of *H. forskåli* was studied by immunofluorescence microscopy. The tubule wall is made up of an outer mesothelium and an inner epithelium encompassing a thick connective tissue sheath [5]. The mesothelium is the tissue layer involved in adhesion. Antibodies raised against the adhesive protein were used to localize the protein in the sections. The strongest immunoreactivity was found in the mesothelium, which was extensively labelled (Figure 7(a)). The sections were counterstained with DAPI (Figures 7(b) and 7(d)) which stains the nuclei. Preimmune serum was used as a negative control (Figure 7(c)).

### 3.6. Immunogold Electron Microscopy

**Figure 8:**
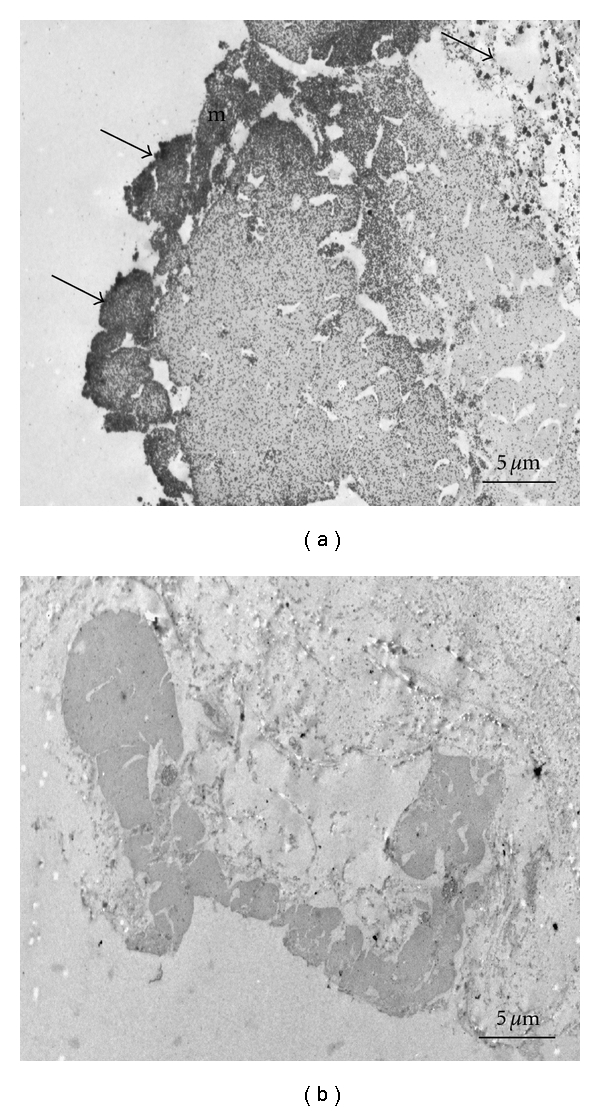
Immunocytochemical localization of adhesive protein in Cuvierian tubules. (a) Sections of Cuvierian tubules incubated with antibody against adhesive protein from Cuvierian tubules (PoAb-Ctub). (b) Sections of Cuvierian tubules incubated with preimmune serum. Nanogold-labeled anti-rabbit IgG was used as secondary antibody. *Arrows*: nanogold particles; *m*: mesothelium.

Transmission electron microscopy showed a strong immunoreactivity in the mesothelium layer and in vacuole cells (darker areas indicated by arrows in Figure 8(a)). Preimmune serum did not show any immunoreactivity (Figure 8(b)).

### 3.7. Adhesion of Cuvierian Tubule Extract to Various Surfaces

#### 3.7.1. Teflon

**Figure 9:**
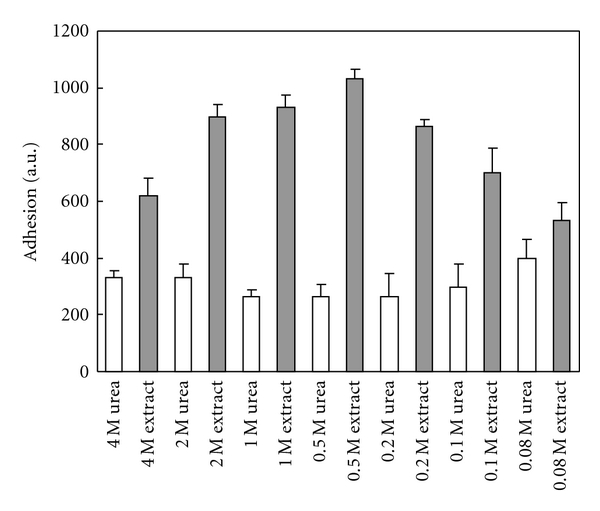
Effect of various concentrations of urea on adhesion to Teflon surface. Results are given in arbitrary units ± SD (*n* = 3).

Cuvierian tubules were extracted with 4 M urea, 0.5 M Tris-HCl pH 7.5. Adhesion was measured after dilution of the extract to various urea concentrations. As a reference, buffers with the same concentration of urea were used. The strongest adhesion to Teflon blocks was obtained at a dilution of extract to 0.5 M urea (767 arbitrary units, after subtraction of adhesion measured with buffer alone [control value]; 0.125 mg/mL of protein) (Figure 9). Higher or lower concentrations of urea resulted in lower adhesion. 

In control experiments, no adhesive properties of BSA (1 mg/mL) in various urea solutions were observed.

**Figure 10:**
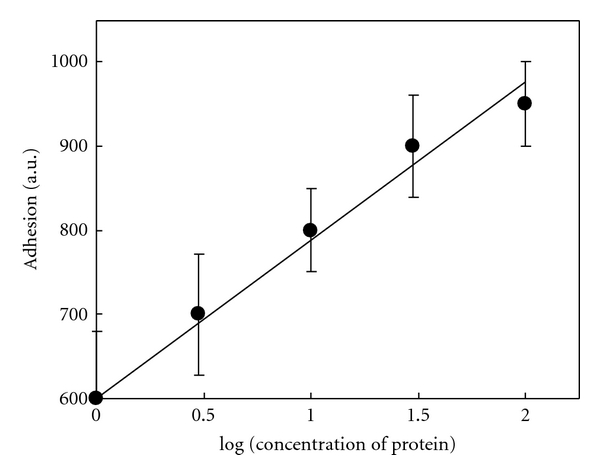
Correlation between various concentrations of protein in logarithmic scale and adhesion (standard curve). The highest concentration measured was 100 *μ*g/mL of protein. Results are given in arbitrary units ± SD (*n* = 3).

A standard curve was obtained with a logarithmic dilution of 0.5 M urea extract (the concentration of urea at which the highest adhesion was observed). Dilution of protein caused a decrease in adhesion (Figure 10).

#### 3.7.2. Glass Surface

Cuvierian tubule extract in 4 M urea, 0.5 M Tris-HCl pH 7.5 buffer was diluted to various concentrations and adhesion to glass blocks was measured. The strongest adhesion was observed at 1 M urea concentration of the Cuvierian tubule extract (366 arbitrary units, after subtraction of control value; 0.25 mg/mL of protein).

#### 3.7.3. Iron Surface

Extract of Cuvierian tubules was diluted to various concentrations of urea and adhesion was measured using two iron blocks. Strong interference of the extraction buffer was observed in almost all urea concentrations. The strongest adhesion was observed with 1 M urea extract (350 arbitrary units, after subtraction of control value; 0.25 mg/mL of protein).

#### 3.7.4. Silicone Surface

Adhesion of various concentrations of Cuvierian tubule extract was measured using two silicone blocks. The strongest adhesion was observed at a concentration of 1 M and 0.5 M urea (316 and 250 arbitrary units, after subtraction of control value; 0.25 mg/mL and 0.125 mg/mL of protein).

#### 3.7.5. Gelatine Surface

Adhesion of various concentrations of Cuvierian tubules extract was measured using blocks covered with gelatine. Measurement was interfered by buffer which also showed strong adhesion. The highest adhesion of the extract was observed with 2 M urea extract (400 arbitrary units, after subtraction of control value; 0.5 mg/mL of protein).

### 3.8. Effect of Metal Ions and EDTA on Adhesion

**Figure 11:**
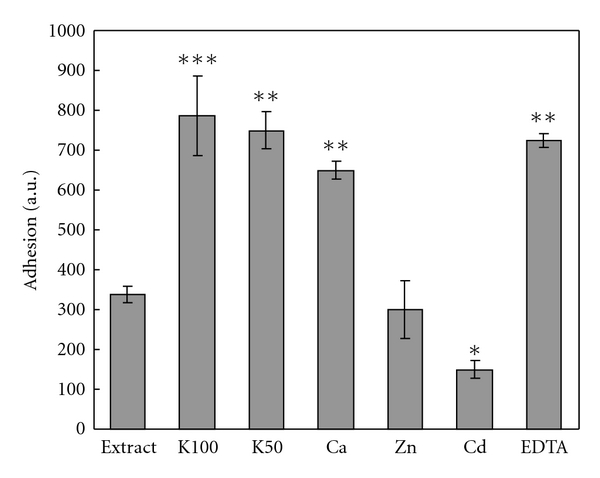
Effect of metal cations and EDTA on adhesion in 0.5 M urea extract. Results are given in arbitrary units ± SD (*n* = 3). Level of significance: *P < 0.05; **P < 0.01; ***P < 0.001.

The adhesive forces of Cuvierian tubule extract were measured in the presence of K^+^ (100 mM and 50 mM), Ca^2+^ (5 mM), Zn^2+^ (5 mM), Cd^2+^(5 mM), and EDTA (10 mM). The results revealed a positive effect on adhesion by potassium and EDTA and a negative effect by cadmium (Figure 11). Zinc did not cause a significant change of adhesion.

### 3.9. Effect of Antibody

**Figure 12:**
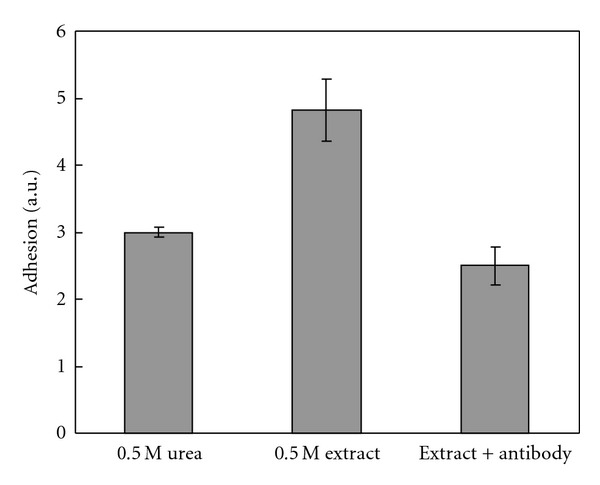
Neutralization of adhesion of *H. forskåli* extract by antibody (PoAb-Ctub). Results are given in arbitrary units ± SD (*n* = 3).

Adhesion could be neutralized by addition of antibody (PoAb-Ctub). Adhesion of neutralized extract decreased in comparison to the nontreated extract in the absence of antibody (Figure 12). The results indicate that the antibody binds to the adhesive protein and inhibits the adhesion. 

Adhesion could also be abolished by treatment of the Cuvierian tubule extract with trypsin solution (not shown).

## 4. Discussion and Conclusions

Investigation of marine adhesives is a challenging task because of their very poor solubility in water [5, 24]. Studies on adhesive proteins from invertebrates hitherto mainly concerned the characterization of permanent adhesives from organisms like mussels and barnacles (e.g., [25, 26]). Great success has been obtained in mussels, where ten proteins are currently known to be involved in adhesion and nine have been actually isolated [7, 27]. Approaches to their biotechnological and biomedical exploitation have been started [28–31]. One of the ten proteins that have been identified to be involved in mussel adhesion is precollagen D that has been found in the distal thread of mussels, which is flanked by a silk-fibroin domain [32]. The adhesives employed by echinoderms are, in contrast, poorly understood. Studies on the adhesive secretion from Cuvierian tubules mainly focused on histological characterization (e.g., [14]); biochemical studies have been performed by Flammang et al. [2] and DeMoor et al. [5]. 

In our approach, we combined the wide knowledge on mussel adhesives and the poorly understood model of *H. forskåli.* Using antibodies against recombinant precollagen D, we could identify a protein involved in adhesion in *H. forskåli.*

The first task was homogenization of Cuvierian tubules which are highly insoluble [5]. In order to solubilize the material several buffers were used. Basic, strong denaturing buffers like 4 M urea, 0.5 M Tris-HCl pH 7.5 gave best results, but some nonsolubilized material still remained. We did not use buffers containing SDS like DeMoor et al. [5] because SDS might interfere with further experiments. 

After extraction we had to prove the presence of adhesive proteins in the solubilized material. One proof to confirm the presence of adhesive proteins was the use of glass beads to isolate adhesive protein. Only proteins with adhesive properties will remain on the beads after several washing cycles. Second, measurement of adhesion of the extract was used as a test for the presence of adhesive protein as well. From these experiments we obtained convincing evidence that the protein which we identified is indeed an adhesive protein.

The reason why Cuvierian tubules are so poorly soluble was not investigated in this study. Aggregation of proteins may be due to the formation of cross-links between proteins composing the adhesive [5] like di-DOPA in mussels [33, 34] and disulfide bonds in barnacles [26, 35]. However, by using the buffer advised by Kamino et al. [26] for barnacles, solubilization was not improved, which may suggest that other cross-links are involved in tubule adhesive aggregation. 

Electrophoretic analysis of tubule print material from Cuvierian tubules done by DeMoor et al. [5] revealed ten different proteins in the range from 17–220 kDa. In our experiments, several proteins in the range from 10–220 kDa were detected in Cuvierian tubule extract. In seminative gels, a reduced number of bands was observed, indicating the presence of conformational oligomers of some of the proteins or the presence of dimers. 

After identification, isolation of the protein was a challenging task. Adhesive proteins from Holothuroidea have not been isolated so far. In this work several methods were tested which had previously been applied for isolation of nonadhesive protein [23, 36, 37] to get a high quality and quantity of the adhesive protein. Electroelution was found to produce the highest quantity of the protein, which then allowed its use for the production of antibodies.

The antibody obtained was used in a further isolation method, immunoprecipitation. From all the methods of isolation, electroelution and gel filtration were found the most useful for other applications.

Using various staining methods, the composition of Cuvierian tubules was analyzed. From sections stained with Cason's trichrome it is possible to conclude that Cuvierian tubules mainly contain collagen. In the mesothelium, cytoplasm of adluminal cell was observed. By staining with hematoxylin and eosin, it was possible to observe collagen which is stained red (eosin). Immunostaining of the sections of Cuvierian tubules with antibodies against adhesive protein from Cuvierian tubules confirmed that the mesothelium is the tissue layer responsible for adhesion as reported by DeMoor et al. [5]. 

To localize more precisely the adhesive protein in the Cuvierian tubules, immunogold labelling and transmission electron microscopy studies were performed. The antisera were strongly immunoreactive in the mesothelium and vacuole, confirming previous studies showing that the adhesive is located in this layer. There was no labelling with the preimmune sera, confirming that the observed immunoreaction is genuinely between a specific antibody and antigen. 

Measurement of adhesion of Cuvierian tubules has previously been studied under various conditions [8, 9, 38], but measurement of the adhesive strength of Cuvierian tubules extract and isolated protein has not been done. A protein which can adhere to different surfaces underwater and at low temperature could have a great potential for application in technology and in antifouling. Therefore, various tests on the adhesion properties of the isolated protein/extract were performed. 

Dalsin et al. [39] and Lee et al. [40] found that mussels can adhere to any organic or inorganic surfaces. In our experiments with Cuvierian tubule extracts from *H. forskåli* not all surfaces were suitable for adhesion. The best results were obtained with Teflon [poly(tetrafluoroethylene)], a hydrophobic polymer. There was a very low adhesion of the extraction buffer and strong adhesion of the extract. By measuring adhesion in various dilutions of Cuvierian tubule extract it was possible to find the urea concentration with the highest adhesion of the adhesive protein and to develop a standard curve for the extract. The highest adhesion was obtained with 0.5 M urea extract; this result is in line with our previous results [8] showing that urea has an impact on adhesion. 

The finding that Teflon shows strong adhesion is thought to be caused by the fact that the adhesive protein is quite hydrophobic (hence its insolubility) and will tend to displace water from the Teflon, resulting in a force (from the water molecules) that will resist water being pulled into the space between the two Teflon blocks and hence producing a “bond” where in consequence water is moving to its lowest possible energy state.

Measurement of adhesion on silicone surface gave similar results to Teflon but adhesion in the presence of buffer alone could be observed. Silicone surface is rougher than Teflon surface, perhaps resulting in a higher bonding energy between the surfaces compared to that of Teflon for both extract and buffer only.

The other tested surfaces (glass, iron, and gelatine) showed strong adhesion in the presence of buffer only, which prevented them from being used as suitable surfaces for measuring adhesion mediated by the Cuvierian tubule extract. 

Comparing Teflon and glass it is possible to conclude that adhesion depends on the surface. If the surface is hydrophobic (Teflon) adhesion is strong and there is no interference of buffer; when surface is hydrophilic (glass) [41] interaction between buffer and surface could be observed.

Studies on the effect of cations and EDTA on adhesion revealed that cadmium inhibited adhesion, while EDTA and potassium increased it. Cadmium inhibits formation of disulfide bonds, possibly resulting in the protein losing its structure and adhesive properties [42]. EDTA is a chelating agent which is widely used to sequester di- and trivalent metal ions; it is possible that it chelates some cations which are involved in adhesion. Potassium affects the solubility of amino acids in aqueous electrolyte solutions [43] which could cause better solubilization of the adhesive protein and increase its adhesive properties, or make protein refold and groups responsible for adhesion more suitable for adhesion. Ionic strength could be involved in increasing adhesion as well. 

The adhesive properties of Cuvierian tubule extract could be neutralized by addition of the antibodies against the adhesive protein, most likely because of the binding of the antibodies to epitopes on the protein molecule that are involved in adhesion or by sterical interference with the adhesion between the protein and the surface. Neutralization of the adhesive protein by trypsin or antibodies could be useful in designing antifouling compounds. Knowledge about blocking adhesion can be helpful to understand adhesive processes and their inhibition if necessary. 

The isolation of the adhesive protein will facilitate the identification of the gene encoding this molecule, which may be used to produce the recombinant protein. This study could help to develop new water-resistant adhesive proteins for (bio)technical and biomedical applications.
